# A logistic regression investigation of the relationship between the Learning Assistant model and failure rates in introductory STEM courses

**DOI:** 10.1186/s40594-018-0152-1

**Published:** 2018-12-28

**Authors:** Jessica L. Alzen, Laurie S. Langdon, Valerie K. Otero

**Affiliations:** University of Colorado Boulder, 249 UCB, Boulder, CO 80309 USA

**Keywords:** Learning assistant, LA, DWF, Retention, Failure, Underrepresented students

## Abstract

**Background:**

Large introductory STEM courses historically have high failure rates, and failing such courses often leads students to change majors or even drop out of college. Instructional innovations such as the Learning Assistant model can influence this trend by changing institutional norms. In collaboration with faculty who teach large-enrollment introductory STEM courses, undergraduate learning assistants (LAs) use research-based instructional strategies designed to encourage active student engagement and elicit student thinking. These instructional innovations help students master the types of skills necessary for college success such as critical thinking and defending ideas. In this study, we use logistic regression with pre-existing institutional data to investigate the relationship between exposure to LA support in large introductory STEM courses and general failure rates in these same and other introductory courses at University of Colorado Boulder.

**Results:**

Our results indicate that exposure to LA support in any STEM gateway course is associated with a 63% reduction in odds of failure for males and a 55% reduction in odds of failure for females in subsequent STEM gateway courses.

**Conclusions:**

The LA program appears related to lower course failure rates in introductory STEM courses, but each department involved in this study implements the LA program in different ways. We hypothesize that these differences may influence student experiences in ways that are not apparent in the current analysis, but more work is necessary to support this hypothesis. Despite this potential limitation, we see that the LA program is consistently associated with lower failure rates in introductory STEM courses. These results extend the research base regarding the relationship between the LA program and positive student outcomes.

## Background

Science, technology, engineering, and mathematics (STEM) departments at institutes of higher education historically offer introductory courses that can serve up to 1000 students per semester. Introductory courses of this size, often referred to as “gateway courses,” are cost-effective due to the number of students able to receive instruction in each semester, but they often lend themselves to lecture as the primary method of instruction. Thus, there are few opportunities for substantive interaction between the instructor and students or among students (Matz et al., 2017; Talbot, Hartley, Marzetta, & Wee, 2015). Further, these courses typically have high failure rates (Webb, Stade, & Grover, 2014) and lead many students who begin as STEM majors to either switch majors or drop out of college without a degree (Crisp, Nora, & Taggart, 2009). In efforts to address these issues, STEM departments across the nation now implement active engagement strategies in their classes such as peer instruction and interactive student response systems (i.e., clicker questions) during large lecture meetings (Caldwell, 2007; Chan & Bauer, 2015; Mitchell, Ippolito, & Lewis, 2012; Wilson & Varma-Nelson, 2016). In addition to classroom-specific active engagement, interventions are programs designed to guide larger instructional innovations from an institution level, such as the Learning Assistant (LA) model.

The LA model was established at University of Colorado Boulder in 2001. The program represents an effort to change institutional values and practices through a low-stakes, bottom-up system of course assistance. The program supports faculty to facilitate increased learner-centered instruction in ways that are most valued by the individual faculty member. A key component of the LA model is undergraduate learning assistants (LAs). LAs are undergraduate students who, through guidance, encourage active engagement in classes. LAs facilitate discussions, help students manage course material, offer study tips, and motivate students. LAs also benefit as they develop content mastery, teaching, and leadership skills. LAs get a monthly stipend for working 10 h per week, and they also receive training in teaching and learning theories by enrolling in a math and science education seminar taught by discipline-based education researchers. In addition, LAs meet with faculty members once a week to develop deeper understanding of the content, share insights about how students are learning, and prepare for future class meetings (Otero, 2015).

LAs are not peer tutors and typically do not work one-on-one with students. They do not provide direct answers to questions or systematically work out problems with students. Instead, LAs facilitate discussion about conceptual problems among groups of students and they focus on eliciting student thinking and helping students make connections between concepts. This is typically done both in the larger lecture section of the course as well as smaller meetings after the weekly lectures, often referred to as recitation. LAs guide students in learning specific content, but also in developing and defending ideas—important skills for higher-order learning in general. The model for training LAs and the design of the LA program at large are aimed at making a difference in the ways students think and learn in college overall and not just in specific courses. That is, we expect exposure to the program to influence student success in college generally.

Prior research indicates a positive relationship between exposure to LAs and course learning outcomes in STEM courses (Pollock, 2009; Talbot et al., 2015). Other research suggests that modifying instruction to be more learner-centered helps to address high failure rates (Cracolice & Deming, 2001; Close, Mailloux-Huberdeau, Close, & Donnelly, 2018; Webb et al., 2014). This study seeks to further understand the relationship between the LA program and probability of student success. Specifically, we answer the following research question: How do failure rates in STEM gateway courses compare for students who do and do not receive LA support in any STEM gateway course? We investigate this question because, as a model for institutional change, we expect that LAs help students develop skills and dispositions necessary for success in college such as higher-order thinking skills, navigating course content, articulating and defending ideas, and feelings of self-efficacy. Since skills such as these extend beyond a single course, we investigate the extent to which students exposed to the LA program have lower failure rates in STEM gateway courses generally than students who are not exposed to the program.

## Literature review

The LA model is not itself a research-based instructional strategy. Instead, it is a model of social and structural organization that induces and supports the adoption of existing (or creation of new) research-based instructional strategies that require increased teacher-student ratio. The LA program is at its core, a faculty development program. However, it does not push specific reforms or try to change faculty directly. Instead, the opt-in program offers *resources and structures* that lead to changes in values and practices among faculty, departments, students, and the institution (Close et al., 2018; Sewell, 1992). Faculty members write proposals to receive LAs (these proposals must involve course innovation using active engagement and student collaboration), students apply to be LAs, and departments match funding for their faculty’s requests for LAs. Thus, the LA program has become a valued part of the campus community.

The body of research that documents the relationship between student outcomes and the LA program is growing. Pollock (2006) provided evidence regarding the relationship between instructional innovation including LAs and course outcomes in introductory physics courses at University of Colorado Boulder by comparing three different introductory physics course models (outlined in Table 1).

**Table 1:** Pollock (2006) Physics I model descriptions

Name	Key traits
#1: University of Washington Physics Tutorials materials (McDermott & Shaffer, 2002) with LAs and TAs Fall 2003; Spring 2004	Trained TAs and LAs facilitated small group work in recitation sections. Students worked on homework assigned specifically for University of Washington Physics Tutorials. TAs and LAs did not provide answers to the homework as much as guided discussion through questioning techniques to help students construct their own knowledge via discussion. TAs and LAs participated in weekly planning meetings to prepare for recitation meetings.
#2: *Physics for Scientists and Engineers* workbook (Knight, 2004) with TAs Fall 2004	TAs facilitated small group work in which students completed exercises in the *Physics for Scientists and Engineers* workbook attached to a course textbook for half of the term. During the last half of the semester, recitation was used to review homework in a more traditional fashion, with TAs directly answering questions from the homework assignments. Training for TAs was much more limited.
#3: *Physics for Scientists and Engineers* workbook with traditional TAs Spring 2005	No use of small group work. Recitation sessions oriented around the TA providing answers to homework exercises rather than students working collaboratively to develop conceptual understanding.

Pollock provides two sources of evidence related to student outcomes regarding the relative effectiveness of these three course models. First, he discussed average normalized learning gains on the force and motion concept evaluation (FMCE; Thornton & Sokoloff, 1998) generally. The FMCE is a concept inventory commonly used in undergraduate physics education to provide information about student learning on the topics of force and motion. Normalized learning gains are calculated by finding the difference in average post-test and pre-test in a class and dividing that value by the difference between 100 and the average pre-test score. It is conceptualized as the amount the students learned divided by the amount they could have learned (Hake, 1998).

Prior research suggests that traditional instructional strategies yield an average normalized learning gain of about 15% and research-based instructional methods such as active engagement and collaborative learning yield on average about 63% average normalized learning gains (Thornton, Kuhl, Cummings, & Marx, 2009). The approach using the University of Washington Tutorials with LAs saw a normalized learning gain of 66% on the FMCE from pre-test to post-test. Average learning gains for the approach using Knight’s (2004) workbooks with TAs were about 59%, and average normalized learning gains for the traditional approach were about 45%. The average normalized learning gains for all three methods in Pollock’s study are much higher than what the literature would expect from traditional instruction, but the course model including LAs is aligned with what is expected from research-based instructional strategies. Second, Pollock further investigated the impact of the different course implementations on higher and lower achieving students on FMCE scores. To do this, he considered students with high pre-test scores (those with pre-test scores > 50%) and students with low pre-test scores (those with pre-test scores < 15%). For both groups of students, the course implementation that included recitation facilitated by trained TAs and LAs had the highest normalized learning gains as measured by the FMCE.

In a similar study at Florida International University, Goertzen et al. (2011) investigated the influence of instructional innovations through the LA program in introductory physics. As opposed to the University of Washington Tutorials in the Pollock (2006) study, the research-based curriculum materials used by Florida International University were Open Source Tutorials (Elby, Scherr, Goertzen, & Conlin, 2008) developed at University of Maryland, College Park. Goertzen et al. (2011) used the Force Concept Inventory (FCI; Hestenes, Wells, & Swackhamer, 1992) as the outcome of interest in their study. Despite the different curriculum from the Pollock (2006) context, Goertzen et al. found that those students exposed to the LA-supported courses had a 0.24 increase in mean raw gain in scores from pre-test to post-test while students in classes that did not include instructional innovations only saw raw gains of 0.16.

In an attempt to understand the broader relationship between the LA program and student outcomes, White et al. (2016) investigated the impacts of the LA model on student learning in physics across institutions. In their study, White et al. used paired pre-/post-tests from four concept inventories (FCI, FMCE, Brief Electricity and Magnetism Assessment [BEMA; Ding, Chabay, Sherwood, & Beichner, 2006], and Conceptual Survey of Electricity and Magnetism [CSEM]) at 17 different institutions. Researchers used data contributed to the Learning Assistant Alliance through their online assessment tool, Learning About STEM Student Outcomes^1^ (LASSO). This platform allows institutions to administer several common concept inventories, with data securely stored on a central database to make investigation across institutions possible (Learning Assistant Alliance, 2018). In order to identify differences in learning gains for students who did and did not receive LA support, White et al. tested differences in course mean effect sizes between the two groups using a two-sample *t* test. Across all of the concept inventories, White et al. found average Cohen’s *d* effect sizes 1.4 times higher for LA-supported courses compared to courses that did not receive LA support.

The research about the LA model shows that students exposed to the model tend to have better outcomes than those in more traditional lecture-based learning environments. However, due to the design of the program and the goals of the LA model, there is a reason to expect that there are implications for more long-term outcomes. LAs are trained to help students develop skills such as developing and defending ideas, making connections between concepts, and solving conceptual problems. Prior research suggests that skills such as these develop higher-order thinking for students. Martin et al. (2007) compared learning outcomes and innovative problem-solving for biomedical engineering students in inquiry-based, active engagement and traditional lecture biotransport courses. They found that both groups reached similar learning gains but that the active engagement group showed greater improvement in innovative thinking abilities. In a similar study, Jensen and Lawson (2011) investigated achievement and reasoning gains for students in either inquiry-based, active engagement or lecture-based, didactic instruction in undergraduate biology. Results indicated that students in active engagement environments outperformed students in didactic environments on more cognitively demanding items, while the groups performed equally well on items requiring low levels of cognition. In addition, students in active engagement groups showed greater ability to transfer reasoning among contexts.

This research suggests that active engagement such as what is facilitated with the LA model may do more than help students gain knowledge in a particular discipline in a particular course. Over and above, active engagement helps learners grow in reasoning and transfer abilities generally. This increase in higher-order thinking may help students to develop skills that extend beyond the immediate course. However, there is only one study focused on the LA model that investigates long-term outcomes related to the program. Pollock (2009) investigated the potential long-term relationship between exposure to the LA program and conceptual understanding in physics. In this line of inquiry, Pollock compared BEMA assessment scores for those upper-division physics majors who did and did not receive LA support in their introductory Physics II course, the course in which electricity and magnetism is first covered. Pollock’s results indicate that those students who received LA support in Physics II had higher BEMA scores following upper-division physics courses than those students who did not receive LA support in Physics II. This research provides some evidence to the long-term relationship between exposure to the LA program and conceptual learning. In the current study, we continue this line of inquiry by investigating the relationship between receiving LA support in a gateway course and the potential relationship to course failure in subsequent gateway courses. This study also contributes to the literature on the LA program as no prior research attempts to examine the relationship between taking LA-supported courses and student outcomes while controlling for variables that may confound this relationship. This study thus represents an extension of the previous work regarding the LA model in terms of both the methodology and the outcome of interest.

## Data

Data for this study come from administrative records at University of Colorado Boulder. We focus on 16 cohorts of students who entered the university as full-time freshmen for the first time each fall semester from 2001 to 2016 and took Physics I/II, General Chemistry I/II, Calculus I/II (Math department), and/or Calculus I/II for Engineers (Applied Math department). The dataset includes information for 32,071 unique students, 23,074 of whom took at least one of the above courses with LA support. Student-level data includes information such as race/ethnicity, gender, first-generation status, and whether a student ever received financial aid. Additional variables include number of credits upon enrollment, high school grade point average (GPA), and admissions test scores. We translate SAT total scores to ACT Composite Scores using a concordance table provided by the College Board to have a common admissions test score for all students (College Board, 2016). We exclude students with no admissions test scores (about 6% of the sample). We also have data on the instructor of record for each course. The outcome of interest in this study is failing an introductory STEM course. We define failing as receiving either a D or an F or withdrawing from the course altogether after the university drop date (i.e., “DFW”).

An important consideration in creating the data set for this study is timing of receiving LA support relative to taking any STEM gateway course. The data begin with all students who took at least one of the courses included in this study. We keep all students who took all of their STEM LA courses either with or without LA support. We also include all students who received LA support in the very first STEM gateway course they took, regardless of if they had LA support in subsequent STEM gateway courses. We would exclude any student who took a STEM gateway course without LA support and then took another STEM gateway course in a subsequent semester with LA support.

This data limitation ensures that exposure to the LA program happened before or at the same time as the opportunity to fail any STEM gateway course. If it were the case that a student failed a STEM gateway course without LA support, say, in their first year and then took LA-supported courses in the second year, this student would be indicated as an LA student in the data, but the courses taken during the first year would not have been affected by the LA program. Students with experiences such as this would misrepresent the relationship between being exposed to the LA program and probability of course failure. Conveniently, there were not any students with this experience in the current dataset. In other words, for every student in our study who took more than one of the courses of interest, their first experience with any of the STEM gateway courses under consideration included LA support if there was ever exposure to the LA program. Although we did not have to exclude any students from our study for timing reasons, other institutions carrying out similar studies should carefully consider such cases when finalizing their data for analysis.

**Fig. 1:**
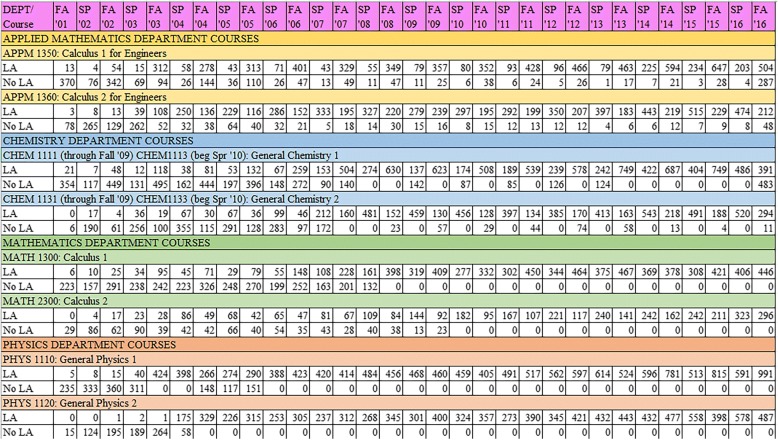
Course enrollment over time by LA exposure

We provide Fig. 1 as a way for readers to gain a better understanding of the adoption of the LA program in each of the departments in this study. This figure also gives information regarding the number of students exposed to LAs or not in each department, course, and term in our study.

## Methods

Ideally, we would design a controlled experiment to estimate the causal effect of LA exposure on the probability of failing introductory STEM courses. To do this, we would need two groups of students: first, those who were exposed to LA support in a STEM gateway course, and second, a comparable group, on average, that significantly differed only in that they were not exposed to LA support in any STEM gateway course. However, many institutions do not begin their LA programs with such studies in mind, so the available data do not come from a controlled experiment. Instead, we must rely on historical institutional data that was not gathered for this type of study. Thus, this study not only contributes to the body of literature regarding the relationship between LA exposure and student outcomes, but it also serves as a model for other institutions with LA programs that would like to use historical institutional data for similar investigations.

### Selection bias

The ways students are assigned to receive LA support in each of the departments represented in this study are not random, and the ways LAs are used in each department are not identical. These characteristics of pre-existing institutional data manifest themselves as issues related to selection bias within a study. For example, in the chemistry department, LA support was only offered in the “on semester” sections of chemistry from 2008 to 2013. “On semester” indicates General Chemistry I in the fall and General Chemistry II in the spring. Thus, there were few opportunities for those students who took the sequence in the “off semester,” or General Chemistry I in the spring and General Chemistry II in the fall to receive LA support in these courses during the span of time covered in this analysis. The most typical reasons why students take classes in the “off semester” are that they simply prioritize other courses more in the fall semester, so there is insufficient space to take General Chemistry I; they do not feel prepared for General Chemistry I in the fall and take a more introductory chemistry class first; or they fail General Chemistry I the first time in the fall and re-take General Chemistry I in the spring. This method of assignment to receiving LA support may overstate the relationship between receiving LA support and course failure in this department. That is, it might be the case that those students who received LA support were those who were more likely to pass introductory chemistry to begin with. Our analysis includes prior achievement variables (described below) to attempt to address these selection bias issues.

In chemistry, LAs attend the weekly lecture meetings and assist small groups of students during activities such as answering clicker questions. Instructors present questions designed to elicit student levels of conceptual understanding. The questions are presented to the students; they discuss the questions in groups and then respond using individual clickers based on their selection from one of several multiple-choice options. LAs help students think about and answer these questions in the large lecture meetings. In addition, every student enrolled in General Chemistry I and II is also enrolled in a recitation section. Recitations are smaller group meetings of approximately 20 students. In these recitation sections, LAs work with graduate TAs to facilitate small group activities related to the weekly lecture material. The materials for these recitation sections are created by the lead instructor for the course and are designed to help students investigate common areas of confusion related to the weekly material.

In the physics and math departments, the introductory courses went from no LA support in any section in any semester to all sections in all semesters receiving LA support. This historical issue affects selection bias in a different way than the off-semester chemistry sequence. One interpretation of decreased course failure rates could be that LA support caused the difference. However, we could not rule out the possibility that failure rates decreased due to other factors that also changed over time. It could be that the university implemented other student supports in addition to the LA model at the same time or that the types of students who enrolled in STEM courses changed. There is no way to determine conclusively which of these (or other) factors may have caused changes in failure rates. Thus, causal estimates of the effect of LA support on failure rates would be threatened by any historic changes that occurred. We have no way of knowing if we might over or underestimate the relationship between LA exposure and course failure rates due to the ways students were exposed (or not) to the LA program in these departments. In order to address this issue, we control for student cohort. This adjustment, described below, attempts to account for differences that might exist among cohorts of students that might be related to probability of failing a course.

The use of LAs in the math department only occurs during weekly recitation meetings. During this weekly meeting, students work in small groups to complete carefully constructed activities designed to enhance conceptual understanding of the materials covered during the weekly lecture. An anomaly in the math department is that though Calculus I/II are considered gateway courses, the math department at this institution is committed to keeping course enrollment under 40. This means that LA support is tied to smaller class sizes in this department. However, since this condition is constant across the timeframe in our study, it does not influence selection bias.

Similar to the math department, the physics department only uses LAs in the weekly recitation meeting. An additional anomaly in physics is that, not incidentally, the switch to the LA model happened concurrently with the adoption of the University of Washington Tutorials in introductory physics (McDermott & Shaffer, 2002). LAs facilitate small group work with the materials in the University of Washington Tutorials during recitation meetings. In other words, it is not possible to separate the effects of the content presentation in the Tutorials from the LAs facilitating the learning of the content in this department. Thus, data from this department might overestimate the relationship between receiving LA support and course failure. However, it should be noted that the University of Washington Tutorials require a low student-teacher ratio, and proper implementation of this curriculum is not possible without the undergraduate LAs helping to make that ratio possible.

Finally, every student in every section of Calculus I and II in the applied math department had the opportunity to be exposed to LA support. This is because LAs are not used in lecture or required recitation meetings, but instead facilitate an additional weekly one-unit course, called workgroup, that is open to all students. Thus, students who sign up for workgroup not only gain exposure to LA support, but they also gain an additional 90 min of time each week formally engaging in calculus material. It is not possible to know if lower failure rates might be due to the additional time on task generally, or exposure to LAs during that time specifically. This might cause us to overestimate the relationship between LA support and course failure. Additionally, those students who are expected to struggle in calculus (based on placement scores on the Assessment and LEarning in Knowledge Spaces [ALEKS] assessment) or are not confident in their own math abilities are more strongly encouraged to sign up for the weekly meeting by their instructors and advisors. Thus, those students who sign up for LA support might be more likely to fail calculus. This might lead us to underestimate the relationship between LA exposure and course failure. Similar to the chemistry department, we use prior achievement variables (described below) to address this issue to the best of our abilities.

We mention one final assumption about the LA model before describing our methods of statistical adjustment. Our data span 32 semesters of 8 courses (see Fig. 1). Although it is surely the case that the LA model adapted and changed in some ways over the course of this time, we make the assumption that the program was relatively stable within department throughout the time period represented in this study.

### Statistical adjustment

Although we do not have a controlled experiment that warrants causal claims, we desire to estimate a causal effect. The current study includes a control group, but it is not ideal because of the potential selection bias in each department described above. However, this study is warranted because it takes advantage of historical data. Our analytic approach is to control for some sources of selection bias. Specifically, we use R to control for standardized high school GPA, standardized admissions test scores, and standardized credits at entry to try and account for issues related to prior aptitude. This helps to address the selection bias issues in the chemistry and applied math departments. Additionally, we control for student cohort to account for some of the historical bias in the physics and math departments. We also control for instructor and course as well as gender (coded 1 = female; 0 = male), race/ethnicity (coded 1 = nonwhite; 0 = white), first-generation status (coded 1 = first-generation college student; 0 = not first-generation college student), and financial aid status (coded 1 = received financial aid ever; 0 = never received financial aid) to disentangle other factors that might bias our results in any department. Finally, we consider possible interaction effects between exposure to LA support and various student characteristics. Table 2 presents the successive model specifications explored in this study. Model 1 controls only for student characteristics. Model 2 adds course, cohort, and instructor factor variables. Model 3 adds an interaction between exposure to the LA program and gender to the model 2 specification.

**Table 2:** Logistic regression model specifications

Model predictor	1	2	3
LA exposure	X	X	X
Female	X	X	X
Nonwhite	X	X	X
First generation	X	X	X
Financial aid recipient	X	X	X
Standardized credits at entry	X	X	X
Standardized HS GPA	X	X	X
Standardized admissions test scores	X	X	X
Course factor		X	X
Cohort factor		X	X
Instructor factor			X
LA exposure-female interaction			X**

**Interactions between LA exposure and nonwhite, first generation, financial aid recipient, standardized HS GPA, and standardized admissions test scores were also tested, but none were found to be statistically significant

The control variables in Table 2 help to account for the selection bias described above as well as other unobserved bias in our samples, but we are limited by the availability of observed covariates. Thus, the results presented here lie somewhere between “true” causal effects and correlations. We know that our results tell us more than simple correlations, but we also know that we are surely missing key control variables that are typically not collected by institutes of higher education such as a measure of student self-efficacy, social and emotional health, or family support. Thus, we anticipate weak model fit, and the results presented here are not direct causal effects. Instead, they provide information about the partial association between course failure and LA support.

We begin our analysis by providing raw counts of failure rates for the students who did and did not receive LA support in STEM gateway courses. Next, we describe the differences between those students who did and did not receive LA support with respect to available covariates. If it is the case that we see large differences in our covariates between the group of students who did and did not receive LA support, we expect that controlling for those factors in the regression analysis will affect our results in meaningful ways. Thus, we close with estimating logistic regression models to disentangle some of the relationship between LA-support and course failure. The variable of most interest in this analysis is the indicator for exposure to the LA program. A student received a “1” for this variable if they were exposed to the LA program either concurrently or prior to taking STEM gateway courses, and a 0 if they took any classes in the study but never had any LA support in those classes.

## Results

Table 3 includes raw pass and failure rates across all courses. Students are counted every time they enrolled in one of the courses included in our study. We see that those students who were exposed to the LA program in at least one STEM gateway course had 6% lower failure rates in concurrent or subsequent STEM gateway course. We also provide the unadjusted odds ratios for ease of comparison with the logistic regression results. The odds ratio represents the odds that course failure will occur given exposure to the LA program, compared to the odds of course failure occurring without LA exposure. Odds ratios equal to 1.0 indicates the odds of failure is the same for both groups. Odds ratios less than 1.0 indicates that exposure to LA support is associated with a lower chance of failing, while odds ratios greater than 1.0 indicates that exposure to LA support is associated with a higher chance of failing. Thus, the odds ratio of 0.65 in Table 3 indicates a lower chance of failure with LA exposure compared to no LA exposure.

**Table 3:** Raw data counts

	Enrolled (*N*)	Pass (*N*)	Fail (*N*)	Fail (%)	Odds ratio
No-LA	16,496	13,144	3352	20	0.65
LA	64,797	55,622	9175	14	
Difference				6	

Although the raw data indicates that students exposed to LA support have lower course failure rates, these differences could be due, at least in part, to factors outside of LA support. To explore this possibility, we next examine demographic and academic achievement differences between the groups. In Table 4, we present the mean values for all of our predictor variables for students who did and did not receive LA support. The top panel presents all of the binary variables, so averages indicate the percentage of students who identify with the respective characteristics. The bottom panel shows the average for the continuous variables. The *p* values are for the comparisons of means from a *t* test across the two groups for each variable. Table 4 indicates that students exposed to the LA program were more likely to be male, nonwhite, non-first-generation students who did not received financial aid. They also had more credits at entry, higher high school GPAs, and higher admissions test scores. These higher prior achievement variables might lead us to think that students exposed to LA support are more likely to pass STEM gateway courses. If this is true, then the relationship between LA exposure and failure in Table 3 may overestimate the actual relationship between exposure to LAs and probability for course failure. Thus, we next use logistic regression to control for potentially confounding variables and investigate any resulting change in the odds ratio.

**Table 4:** Descriptive statistics

	Non-LA (%)	LA (%)	*p* value
Female	45	35	< 0.01
Nonwhite	24	27	< 0.01
First gen	18	16	< 0.01
Financial aid	48	46	0.02
	Mean (SD)	Mean (SD)	
Credits at entry	7 (11)	9 (12)	< 0.01
HS GPA	3.61 (0.35)	3.68 (0.34)	< 0.01
Test score	26 (4)	27 (4)	< 0.01
*N*	8997	23,074	

R calculates logistic regression estimates in logits, but these estimates are often expressed in odds ratios. We present abbreviated logit estimates in the Appendix and abbreviated odds ratios estimates in Table 5. Estimates for all factor variables (i.e., course, cohort, and instructor) are suppressed in these tables for ease of presentation. In order to make the transformation from logits to odds ratios, the logit estimates were exponentiated to calculate the odds ratios presented in Table 5. For example, the logit estimate for exposure to LA in model 1 from the Appendix converts to the odds ratio estimate in Table 5 by finding exp(− 1.41) = 0.24.

**Table 5:** Logistic regression estimates in odds ratios with confidence intervals

	Dependent variable		
	Failed (= 1)		
	(1)	(2)	(3)
LA exposure	0.244*** (0.237, 0.251)	0.411*** (0.381, 0.443)	0.367*** (0.337, 0.400)
Female	0.558*** (0.536, 0.581)	1.132*** (1.079, 1.188)	0.912* (0.835, 0.997)
Nonwhite	0.868*** (0.828, 0.909)	1.096*** (1.043, 1.152)	1.096*** (1.043, 1.151)
First generation	1.173*** (1.110, 1.240)	1.350*** (1.275, 1.428)	1.351*** (1.277, 1.430)
Financial aid recipient	0.568*** (0.547, 0.590)	1.050* (1.004, 1.098)	1.050* (1.004, 1.098)
Credits at entry	0.888*** (0.865, 0.911)	0.786*** (0.762, 0.811)	0.786*** (0.761, 0.810)
HS GPA	0.681*** (0.667, 0.694)	0.569*** (0.557, 0.582)	0.569*** (0.557. 0.582)
ACT	0.760*** (0.742, 0.778)	0.794* (0.773, 0.814)	0.793*** (0.773, 0.814)
LA exposure-female interaction			1.346*** (1.215, 1.491)
Observations	75,563	75,563	75,563
Log likelihood	− 32,462.050	− 28,672.970	− 28,656.720
Akaike Inf. Crit.	64,940.100	57,949.940	57,919.430

Models 2–3 suppress course, cohort, and instructor factor variables

*Note*: *p < 0.05; **p < 0.01; ***p < 0.001

We start off by discussing the results for model 3 as it is the full model for this analysis. Discussion of models 1 and 2 are saved for the discussion of model fit below. The results in model 3 provide information about what we can expect, on average, across all courses and instructors in the sample. We include confidence intervals with the odds ratios. Confidence intervals that include 1.0 suggest results that are not statistically significant (Long, 1997). The odds ratio estimate in Table 5 for model 3 is 0.367 for LA exposure with a confidence interval from (0.337–0.400). Since the odds ratio is less than 1.0, LA exposure is associated with a lower probability of failing, on average, and the relationship is statistically significant because the confidence interval does not include 1.0. Compared to the odds ratio in Table 3 (0.65), these results indicate that covariate adjustment has a large impact on this odds ratio. Failure to adjust for possible sources of confounding variables lead to an understatement of the “effect” of exposure to the LA program on course failure.

Our results show that LA exposure is associated with lower odds of failing STEM gateway courses. We also see that the interaction between exposure to the LA program and gender is statistically significant. The odds ratio of 0.37 for exposure to LA support in Table 5 is for male students. In order to find the relationship for female students, we must exponentiate the logit estimates for exposure to the LA program, female, and the interaction between the two variables (i.e. exp[01.002–0.092 + 0.297] = 0.45; see the Appendix). This means that the LA program actually lowers the odds of failing for male students slightly more than female students. Recall that Table 3 illustrated that the raw odds ratio for failure when exposed to LA support was 0.65. Our results show that after controlling for possibly confounding variables, the relationship between LA support and odds of course failure is better for both male (0.37) and female (0.45) students.

## Discussion and limitations

Throughout this paper, we have been upfront about the limitations of the current analysis. Secondary analysis of institutional data for longstanding programs is complex and difficult. In this penultimate section, we mention a few other limitations to the study as well as identify some ideas for future research that could potentially bolster the results found here or identify where this analysis may have gone astray.

First, and most closely related to the results presented above is model fit. The McFadden pseudo R-squared (Verbeek, 2008) values for the three models are 0.0708, 0.1793, and 0.1797 respectively. These values indicate two things: (1) that the data do not fit any of the models well and (2) that the addition of the interaction term does little to improve model fit. This is also seen in the comparison of AIC and log likelihood values in Table 5. We spend significant time on the front end of this paper describing why these data are not ideal for understanding the relationship between exposure to the LA program and probability of failing, so we do not spend additional time here discussing this lack of goodness-of-fit. Instead, we acknowledge this as a limitation of the current analysis and reiterate the desire to conduct a similar type analysis to what is presented here with data more likely to fit the model. Such situations would include institutions that have the ability to compare, for example, large samples of students with and without LA exposure within the same semester, course, and instructor. Another way to improve such data would be to include a way to control for student confidence and feelings of self-efficacy. For example, the descriptions of selection bias above indicate that students in Applied Math might systematically be students who differ in terms of self-confidence. Data that could control for such factors would better facilitate understanding of the relationship between exposure to LA support and course failure. Alternatively, it may be more appropriate to consider the nested structure of the data (i.e., students nested within courses nested within departments) in a context with data better suited for such analysis. Hierarchical linear modeling might even be appropriate for a within-department study if it would be reasonable to consider students nested within classes if there was sufficient sample size at the instructor level.

Second, in addition to a measure of student self-efficacy, there are other variables that might be interesting to investigate such as transfer, out-of-state, or international student status; if students live on-campus; and a better measure of socioeconomic status than receiving financial aid. These are other important student characteristics that might uncover differential relationships between the LA program and particular types of students. Such analysis is important because persistence and retention in gateway courses—particularly for students from traditionally marginalized groups—are an important concern for institutions generally and STEM departments specifically. If we are to maintain and even build diversity in these departments, it is crucial we have solid and clear work in these areas.

Third, although this study controls for course- and instructor-level factors, there are surely complications introduced into this study due to the differential way the LA program is implemented in each department. A more careful study within department is another interesting and valuable approach to understanding the influence of the LA program but one that this data is not well-suited for. Again, there is a need for data which includes students exposed to the LA program and not exposed within the same term, course, and instructor to better disentangle the relationship. Due to the nature of the way the LA program was taken up at University of Colorado Boulder, we do not have the appropriate data for such an analysis.

Finally, an interesting consideration is the choice of outcome variable made in this analysis. Course failure rates are particularly important in gateway courses because failing such a course can lead students to switch majors or drop out of college. We do see a relationship between the LA model and lower failure rates in the current analysis. However, other approaches to course outcomes include course grades, pass rates, average GPA in other courses, and average grade anomaly (Freeman et al., 2014; Haak et al., 2011; Matz et al., 2017; Webb, Stade, & Grover, 2014). Similar investigations to what is presented here with other course outcomes are also of interest. For example, course grades would provide more nuanced information regarding how the LA model influences student outcomes. A measure such as Matz et al.’s (2017) average GPA in other courses could provide more information about how the LA program impacts course other than the ones in which the LA exposure occurred. In either of these situations, it would be interesting to see if the LA program would continue to appear to have a greater impact for male students than female. In short, there are a wide variety of student outcomes that have yet to be fully investigated with data from the LA model and more nuanced information would be a valuable contribution to the research literature.

## Conclusion

In this study, we attempt to disentangle the relationship between LA support and course failure in introductory STEM courses. Our results indicate that failure to control for confounding variables underestimates the relationship between exposure to the LA program and course failure. The results here extend the prior literature regarding the LA model by providing evidence to suggest that exposure to the program increases student outcomes in subsequent as well as current courses. Programs such as the LA model that facilitate instructional innovations where students are more likely to be successful increase student retention.

Preliminary qualitative work suggests potential hypotheses for the relationship between LA support and student success. Observations of student-LA interactions indicate that LAs develop safe yet vulnerable environments necessary for learning. Undergraduates are more comfortable revealing their thinking to LAs than to TAs and instructors and are therefore better able to receive input about their ideas. Researchers find that LAs exhibit pedagogical skills introduced in the pedagogy course and course experience that promote deep understanding of relevant content as well as critical thinking and questioning needed in higher education (Top, Schoonraad, & Otero, 2018). Also, through their interactions with LAs, faculty seem to be learning how to embrace the diversity of student identities and structure educational experiences accordingly. Finally, institutional norms are changing as more courses adopt new ways of teaching students. For example, the applied math department provides additional time on task because of the LA program. Although we do not know if it is the additional time on task, the presence of LAs, or a combination of both that drives the relationship between LA exposure and lower course failure rates, both the additional time and LA exposure occur because of the LA program generally.

Further work is necessary to more fully understand the relationship between the LA program and student success. Although we controlled for several student-level variables, we surely missed key variables that contribute to these relationships. Despite this limitation, the regression analysis represents an improvement over unadjusted comparisons. We used the available institutional data to control for variables related to the selection bias present in each department’s method of assigning students to receive LA support. More research is needed to identify if the emerging themes in the present study are apparent at other institutions. Additional research with data better suited to isolate potential causal effects is also needed to bolster the results presented here. Despite the noted limitations discussed here, the current findings are encouraging for further development and implementation of the LA program in STEM gateway courses. Identifying relationships between models for change and lower course failure rates are helpful for informing future decisions regarding those models.

## Appendix

**Table 6:** Logistic regression estimates in logits

	Dependent variable		
	Failed (= 1)		
	(1)	(2)	(3)
LA exposure	− 1.410*** (0.015)	− 0.889*** (0.039)	− 1.002*** (0.043)
Female	− 0.583*** (0.021)	0.124*** (0.025)	− 0.092** (0.045)
Nonwhite	− 0.142*** (0.024)	0.092*** (0.025)	0.092*** (0.025)
First generation	0.160*** (0.028)	0.300*** (0.029)	0.301*** (0.029)
Financial aid recipient	− 0.565*** (0.020)	0.049** (0.023)	0.049** (0.023)
Credits at entry	− 0.119*** (0.013)	− 0.241*** (0.016)	− 0.241*** (0.016)
HS GPA	− 0.385*** (0.010)	− 0.564*** (0.011)	− 0.564*** (0.011)
ACT	− 0.274*** (0.012)	− 0.231*** (0.013)	− 0.232*** (0.013)
LA exposure*female			0.297*** (0.052)
Observations	75,563	75,563	75,563
Log likelihood	− 32,462.050	− 28,672.970	− 28,656.720
Akaike Inf. Crit.	64,940.100	57,949.940	57,919.430

Models 2–3 suppress course, cohort, and instructor factor variables

*Note:***p* < 0.1; ***p* < 0.05; ****p* < 0.01

## Abbreviations

BEMA: Brief Electricity and Magnetism Assessment
CSEM: Conceptual Survey of Electricity and Magnetism
FCI: Force Concept Inventory
FCME: Force and Motion Concept Evaluation
LA model: Learning Assistant model
LAs: Learning assistants
PLTL: Peer-led team learning
STEM: Science, technology, engineering, and mathematics

## Footnotes

1 For more information about joining LASSO and resources available to support LA programs, visit https://www.learningassistantalliance.org/
